# Current findings and gaps in early initiation of breastfeeding practices in sub-Saharan African countries: A scoping review

**DOI:** 10.7189/jogh.13.04036

**Published:** 2023-05-12

**Authors:** Mari Shimizu, Yoshinori Nakata, Kenzo Takahashi

**Affiliations:** Teikyo University Graduate School of Public Health, Teikyo University, Tokyo, Japan

## Abstract

**Background:**

Early initiation of breastfeeding (EIBF) plays an important role in reducing neonatal and infant mortality. Sub-Saharan African countries have high rates of neonatal and infant mortality, as well as a low prevalence of EIBF. By conducting a scoping review, we aimed to determine the gaps and current understandings of EIBF in Sub-Saharan Africa.

**Methods:**

We conducted this scoping review following the Preferred Reporting Items for Systematic Reviews and Meta-Analyses Extension for Scoping Reviews (PRISMA-ScR) reporting guidelines, focusing on primary studies published from 2008 to 2021. We reviewed their titles and abstracts against the eligibility criteria, selecting the relevant ones to this study’s criteria for a further full-text review.

**Results:**

The identified determinants can be categorized into household, maternal, and health service characteristics. Regarding health service characteristics, many studies reported that cesarean section was strongly associated with the delayed initiation of breastfeeding. Institutional delivery and delivery by skilled birth attendants were also reported to be associated with the early initiation of breastfeeding. Others pointed out that place of residence and wealth index as two household characteristics that were strongly associated with EIBF, as well as maternal characteristics such as older maternal age and higher education status.

**Conclusions:**

We found that only eleven studies on the early initiation of breastfeeding have been conducted in Central and West Africa. Household, maternal, and health service factors have been reported to be associated with the early initiation of breastfeeding across many countries. More studies are needed to fill the current geographic gaps and investigate determinants that have not been examined. Future research should also cover interventions that have been effective in improving EIBF for women after caesarean sections in sub-Saharan Africa. Interventions that promote institutional delivery and deliveries by skilled attendants have the potential to improve the practice.

The early initiation of breastfeeding (EIBF) has been recognized for its health benefits to mothers and newborns. It is defined as the initiation of breastfeeding within the first hour after birth. The World Health Organization (WHO) recommends supporting mothers with initiating breastfeeding after delivery [1]. It is well-known that EIBF contributes to the reduction of child morbidity and mortality by protecting newborns from common childhood illnesses, such as diarrhoea and pneumonia [2]. The Baby-Friendly Hospital Initiative (BFHI) was created by the WHO and UNICEF in 1991, introducing a set of policies and procedures which has since been implemented to facilitate better maternal and neonatal services in health facilities to improve breastfeeding practices in 10 steps, including EIBF in step 4 [3].

Current evidence suggests that EIBF decreases neonatal mortality and early infant mortality [4] by as much as 22% [5]. At 52 infant deaths per 1000 live births, sub-Saharan African countries have the highest rate of infant mortality, significantly above the global average of 28 per 1000 live births [6]. Since the prevalence of EIBF, particularly in sub-Saharan African countries is still low, it could potentially be improved to reduce infant mortality. Issaka et al. [7] indicated that the prevalence of EIBF varies in many sub-Saharan African countries, with the WHO’s EIBF guideline for the rating [8] categorising most as having a “fair” prevalence, equating to a coverage of 30%-49%. The global average prevalence of EIBF is 42% [9]. The coverage was reported to be 40% in West and Central Africa and 65% in Eastern and Southern Africa [9]. The prevalence of EIBF in Central Africa is particularly low, at 37.84% [10]. Considering the high infant mortality rate in the region, the coverage should be further improved to “Good” (50%-89%) and “Very Good” (90%-100%). Understanding of what is known about EIBF in those countries is crucial for decreasing infant mortality rates.

We conducted this scoping review to rapidly map out the current knowledge on and gaps in EIBF sub-Saharan African countries to understand the factors and challenges influencing it.

## METHODS

### Protocol and registration

We conducted this scoping review following the Preferred Reporting Items for Systematic Reviews and Meta-Analyses Extension for Scoping Reviews (PRISMA-ScR) reporting guidelines [11].

### Eligibility criteria

To be included, the study had to be a primary study in English, published from 2008 to 2021, and conducted in sub-Sahara Africa without targeting other countries, while reporting EIBF as either the cause or the outcome. We set 2008 as the lower limit for the criteria since one of the first primary studies on the effect of EIBF practices on neonatal mortality was published in 2007 and conducted Ghana [12]. This study highlighted the opportunity for EIBF to improve neonatal mortality globally.

### Information sources and search

We searched PubMed and Cochrane Library using the terms “Early initiation of breast feeding” AND “Africa” OR “Sub-Saharan Africa”, following the population, concept, and contex (PCC) framework, which we narrowed down before the scoping review (**Table 1**).

**Table 1:** The PCC framework of this study

PCC element	Definition
Population	Women with a live birth /newborn; deliveries at health facilities and communities
Concept	“Provision of mother's breast milk to infants within one hour of birth”; EIBF as study “outcome”
Context	Sub-Saharan African countries, 2008-2021, mother/household/health workers’ perspective

PCC – population (or participants), concept, and context, EIBF – early initiation of breastfeeding

### Selection of sources of evidence

We exported the retrieved studies into EndNote and removed any duplicates. We reviewed their titles and abstracts against the eligibility criteria, selecting the relevant ones for a further full-text review.

### Data charting

We extracted the following key information from the selected studies: 1) author(s), 2) year of publication, 3) origin (source of the country), 4) purpose, 5) target population, 6) methodology, 7) intervention type, 8) outcome, and 9) key findings relevant to this scoping review.

## RESULTS

### Selection of sources of evidence

**Figure 1:**
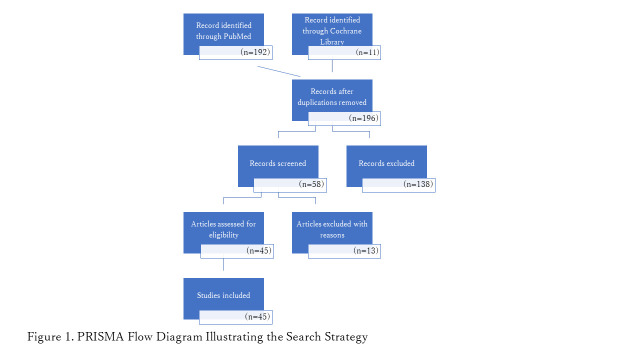
PRISMA flow diagram illustrating the search strategy.

We identified 203 studies (PubMed = 192, Cochrane library = 11) conducted between 2008 and 2021. After deduplication, 196 studies remained. We excluded 138 articles during the title and abstract screening; the remaining fifty-eight articles met the initial eligibility criteria for the full-text review. Forty-five that investigated EIBF and its outcomes in sub-Saharan African countries were then included in the final analysis (**Figure 1**).

### Characteristics of sources of evidence

**Figure 2:**
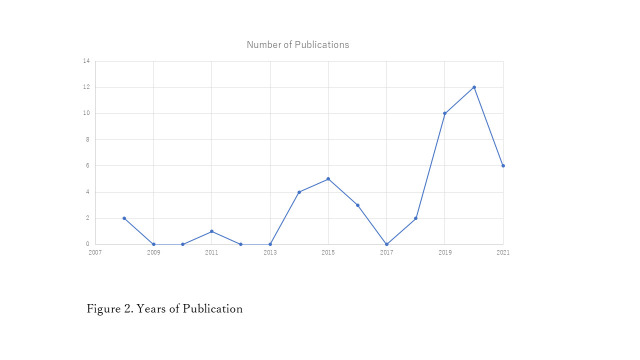
Years of publication.

We categorized the African countries into geographic regions based on the United Nations (UN) statistics classification. A variety of articles were published, particularly from 2019 to 2021 (**Figure 2**). Approximately 64.4% of the studies used data from East African countries and 28.9% used data from West Africa (**Table 2**). There were only three studies from Southern Africa and none from Central African countries. There were 29 studies on EIBF in East African countries; approximately half were on Ethiopia. Thirteen studies examined EIBF in West Africa, half of which covered Ghana or Nigeria.

**Table 2:** Characteristics of included studies

Region	Country	Total number of studies	% by country	% by region	Quantitative	Qualitative	Mixed
East	Ethiopia	14	31.1%	64.4%	14	0	0
	Kenya	2	4.4%		2	0	0
	Malawi	4	8.9%		4	0	0
	Rwanda	1	2.2%		0	1	0
	South Sudan	1	2.2%		1	0	0
	Tanzania	3	6.7%		2	1	0
	Uganda	2	4.4%		1	0	1
	Zimbabwe	2	4.4%		2	0	0
West	Benin	1	2.2%	28.9%	1	0	0
	Ghana	6	13.3%		5	1	1
	Niger	1	2.2%		1	0	0
	Nigeria	5	11.1%		4	0	1
Southern	Namibia	1	2.2%	6.7%	1	0	0
	South Africa	2	4.4%		2	0	0

Most studies involved a secondary data analysis of cross-sectional studies; 45.7% (n = 21) used data from the Demographic and Health Surveys (DHS). Among these, 89.4% (n = 40) were quantitative studies and 74.4% (n = 32) used logistic regression for the data analysis (**Table 2**). A total of two mixed-method studies and three qualitative studies were also published from 2008 to 2021.

### Quantitative studies

Six studies examined the effect of interventions to improve EIBF, while four looked at BFHI. Thirty-one studies focused on finding the determinants of EIBF. **Table 3** summarizes the determinants of EIBF. Most articles studied the determinants of EIBF and factors associated with EIBF comprising the household, mother, and health services.

**Table 3:** Reported determinants of EIBF practices

Categories	Sub-categories	Number of studies	Study reference
Household	Residence	11	[13-23]
	Wealth	5	[13,14,17,19,24]
Mother	Age	4	[17,25-27]
	Education	4	[13,23,28,29]
	Knowledge	4	[24,30-32]
Health service	Mode of delivery	14	[14-16,19,21,25,27-29,32-36]
	Place of delivery	8	[19,22,24,32,34,35,37]
	Support during delivery	7	[14,15,17,18,21,38,39]
	ANC	8	[16,17,22,23,27,29,34,40]

EIBF – early initiation of breastfeeding, ANCD – antenatal care

#### Household characteristics

Several studies evaluated the association of EIBF with household characteristics, including place of residence and wealth. Eleven studied the residence/geographic areas of the households. Some studies mentioned geographic areas, including regions and country districts [13-23], while others studied the difference in the prevalence of EIBF in urban and rural areas [17,19,20,23]. Some studies reported that residing in urban areas was associated with delayed initiation of breastfeeding, while others identified regional disparity in terms of EIBF.

Another household characteristic that was studied was the wealth index. Five studies identified that a higher wealth index was associated with timely EIBF practices [13,14,17,19,24]. Studies in Ethiopia and Nigeria showed that children born in wealthier households have an increased likelihood of EIBF, while it was positively associated with the poorer wealth index households in Namibia. Others found that ethnicity or access to media (eg, radio or TV) were correlated with timely EIBF practices. Mothers with access to media in Ghana and Ethiopia were identified as having an increased likelihood of timely EIBF practices. Some local ethnicity was associated positively with EIBF in Ghana and Nigeria.

#### Maternal characteristics

Maternal characteristics were categorized into maternal age, educational status, and knowledge on EIBF. Three studies found that older maternal age was associated with EIBF [25-27], highlighting that adolescent or teenage mothers were a risk factor for delayed initiation of breastfeeding. Four studies found that higher maternal education has been associated with higher odds of EIBF [13,23,28,29], while another four reported that maternal knowledge played an important role in EIBF practice [24,30-32].

#### Health service characteristics

The association between health services and EIBF practice were categorized as follows: mode of delivery, place of delivery, type of professional support during delivery, and antenatal care (ANC). Fourteen studies examined the mode of delivery; caesarean sections were found to be associated with higher odds of delayed initiation of breastfeeding across different countries [14-16,19,21,25,27-29,32-36]. Seven studies reported that institutional delivery was associated with higher odds of EIBF [19,22,24,32,34,35,37]. Other studies identified that deliveries by skilled birth attendants were positively associated with EIBF [14,15,17,18,21,38]. Haile et al. [18] pointed out that the type of birth attendant played an important role in EIBF, particularly in rural areas. EIBF practice was reported to also be associated with antenatal care (ANC) visits. Eight studies found that a higher number of ANC visits and counselling during ANC visits were associated with EIBF practices [16,17,22,23,27,29,34,40].

#### Other characteristics

Compared to household characteristics, maternal characteristics, and health service factors, analyses of paternal characteristics associated with EIBF were uncommon, as they were examined in only three studies. They reported that maternal education status, religious views, and support of EIBF were associated with EIBF practice. The prevalence of EIBF was higher among mothers with higher education. Delayed EIBF was associated with households that believe in Coptic Orthodoxy in Ethiopia. The studies in Zimbabwe, Ethiopia, and Namibia found that deliveries assisted by skilled health professionals were positively associated with a higher prevalence of timely EIBF practice. The study in Ghana highlighted that the types of health professionals who assisted deliveries in rural areas positively affected the prevalence of EIBF. Timely EIBF practice was lower for those who received delivery assistance from health professionals, such as village health volunteers, traditional health practitioners, and doctors. Ten studies found that a child’s health status was positively associated with EIBF practices. This included sex and birth order. Woldemanuel et al. [23] found that female children had higher odds of EIBF while Ayalew et al. [32] reported that a female child was associated with the late initiation of breastfeeding. First-born children seemed to have lower odds of EIBF.

### Qualitative and mixed-method studies

Three qualitative studies were conducted from 2008 to 2021 in Ghana, Rwanda, and Tanzania [41-43]. They collected data from relatively wide range of stakeholders, including mothers, health workers, policymakers, fathers, and grandmothers. In qualitative studies including mothers, support from family and health workers were identified as facilitators of EIBF. Qualitative studies from decision-makers found that they focused more on exclusive breastfeeding than EIBF. Two mixed methods studies were conducted in Uganda and Nigeria to identify reasons for delaying the initiation of breastfeeding [44,45]. Mothers reported barriers to EIBF practices, including the attitude of birth attendants and the room setting in health facilities. Others mentioned their health conditions, including caesarean section, human immunodeficiency virus (HIV) status, and their beliefs as reasons for delayed initiation of breastfeeding.

## DISCUSSION

### Summary of evidence

With this scoping review, we aimed to map the current findings of studies on EIBF in sub-Saharan African countries. We observed significant geographic disparities among the included studies, as few were conducted in Central, Southern, and West Africa in comparison to East Africa. Considering the low prevalence of EIBF in Central African Countries, more studies are needed in this region.

Most studies employed a quantitative design; there were few qualitative or mixed-method studies. While many studies were conducted using DHS data to investigate the determinants of EIBF practices, few studies used data from interventions. Qualitative studies can report the barriers that cannot be covered by quantitative data. Studies on how mothers, fathers, and health workers feel about EIBF practices can significantly contribute to improving the prevalence of EIBF.

Among the studies that investigated the determinants of EIBF using DHS data, many reported that household characteristics, such as place of residence and wealth index, were associated with EIBF in many countries. Maternal characteristics, including age, education, and knowledge of EIBF were also reported to be relevant to EIBF. Additionally, four factors in health service that were reported to be associated with EIBF were the mode of delivery, place of delivery, professional support during delivery, and ANC. In comparison to household, maternal, and health service characteristics, few studies reported on the paternal and health worker characteristics associated with EIBF. These factors should be studied further, particularly in countries where the prevalence of EIBF is lower, not only with quantitative methods but also with qualitative or mixed study methods.

Since many studies reported that caesarean section is associated with the risk of delayed initiation of breastfeeding practices, further studies should be conducted to investigate the practices that have been implemented to improve EIBF practices for mothers after caesarean section delivery.

Regarding interventions, particularly in countries where the prevalence of EIBF is an issue, those that facilitate ANC visits, delivery with skilled attendants, and institutional delivery may contribute to improving EIBF practices. Interventions can also target areas that were associated with the delayed initiation of breastfeeding.

### Limitations

We did not critically evaluate the selected studies, including their quality or possible bias, as it is not required according to the PRISMA-ScR guidelines. Another limitation is the coverage of a low number of studies due to the criterion for including only primary studies in English. However, this is considered acceptable, as a scoping review only aims to map the current knowledge on a topic.

## CONCLUSIONS

This scoping review has several key findings. First, we found that EIBF has been more frequently studied in East African than in sub-Saharan African countries. There were no studies from Central Africa from 2008 to 2021, although the prevalence of EIBF in the region is very low. We also found that various determinants of EIBF were obtained from studies using DHS data (eg, household, maternal, and health service characteristics). However, few studies examined the impact of interventions on EIBF. Some determinants of EIBF, such as the mode of delivery, have been studied since 2008; however, none of the included studies examined further actions taken to improve EIBF delivery modes that were associated with a higher risk of delayed initiation of breastfeeding.

Further research should be conducted, particularly for countries with a low prevalence of EIBF. Furthermore, various factors that may be associated with EIBF practice should be investigated, including paternal and health worker factors. More research is needed on the results of interventions to improve EIBF in sub-Saharan countries. Interventions to improve EIBF should be tailored to capture the identified determinants of EIBF practices. Encouraging ANC visits, delivery by skilled birth attendants, and institutional delivery may further be emphasized.
